# Eye movements in interception with delayed visual feedback

**DOI:** 10.1007/s00221-018-5257-8

**Published:** 2018-04-19

**Authors:** Clara Cámara, Cristina de la Malla, Joan López-Moliner, Eli Brenner

**Affiliations:** 1 Vision and Control of Action (VISCA) Group, Department of Cognition, Development and Psychology of Education, Institut de Neurociències, Universitat de Barcelona, Barcelona, Spain; 2 Faculty of Behavioural and Movement Science, Vrije Universiteit Amsterdam, Amsterdam, The Netherlands

**Keywords:** Gaze, Pursuit, Visuomotor adaptation, Delay, Motor control

## Abstract

The increased reliance on electronic devices such as smartphones in our everyday life exposes us to various delays between our actions and their consequences. Whereas it is known that people can adapt to such delays, the mechanisms underlying such adaptation remain unclear. To better understand these mechanisms, the current study explored the role of eye movements in interception with delayed visual feedback. In two experiments, eye movements were recorded as participants tried to intercept a moving target with their unseen finger while receiving delayed visual feedback about their own movement. In Experiment 1, the target randomly moved in one of two different directions at one of two different velocities. The delay between the participant’s finger movement and movement of the cursor that provided feedback about the finger movements was gradually increased. Despite the delay, participants followed the target with their gaze. They were quite successful at hitting the target with the cursor. Thus, they moved their finger to a position that was ahead of where they were looking. Removing the feedback showed that participants had adapted to the delay. In Experiment 2, the target always moved in the same direction and at the same velocity, while the cursor’s delay varied across trials. Participants still always directed their gaze at the target. They adjusted their movement to the delay on each trial, often succeeding to intercept the target with the cursor. Since their gaze was always directed at the target, and they could not know the delay until the cursor started moving, participants must have been using peripheral vision of the delayed cursor to guide it to the target. Thus, people deal with delays by directing their gaze at the target and using both experience from previous trials (Experiment 1) and peripheral visual information (Experiment 2) to guide their finger in a way that will make the cursor hit the target.

## Introduction

Exposure to devices in which there is a delay between one’s actions and the consequences of those actions is becoming more and more common. This is because the control of many devices is no longer mechanical, but is mediated by electronic circuits, sometimes involving quite complex computations and transfer of information. The most obvious current example is the circuit in smartphones and tablets. It can take between 50 and 200 ms to update such devices’ displays in response to touch, and these delays can reduce the sensation of direct physical control (Ng et al. 2012). The delay is especially obvious when dragging visible objects across the screen with the finger and object both visible. Even for a slow movement (10 cm/s) and a short delay (50 ms), one will see the finger and object shift relative to each other (for these values you will see a 5 mm displacement). The delays arise because your smartphone takes time to register that the screen has been touched, time for the touch to be interpreted and for the graphics to be adapted accordingly, and time for the adapted graphics to be presented. In most cases, small delays such as the normal delay between a hand moving a computer mouse and the resulting motion of the cursor on the screen are not disturbing. However, longer delays may require adaptation before one can meaningfully use the available feedback to successfully control the device. People can cope with quite long delays. They can intercept a target with a cursor that is delayed by 200 ms, or maybe even more, even when the movement that is made to do so only takes about half a second, and even after very limited exposure to the delay (de la Malla et al. 2014; Honda et al. 2012). Understanding how people deal with delays might, therefore, provide valuable insight into how movements are controlled. Here we examine the role that eye movements might play in controlling a device when the visual feedback of the finger’s movement is delayed.

When performing many everyday activities that involve reaching out for objects, gaze is directed ahead of the arm, presumably to provide information that is needed to guide the movement of the arm (Hayhoe and Ballard 2005; Land and Hayhoe 2001; Mennie et al. 2007; Voudouris et al. 2012, 2016). The precise timing of movements of the eyes with respect to those of the arm may be particularly important in interception (Binsted et al. 2001). Some studies on sports such as cricket (Land and McLeod 2000), racquetball (Diaz et al. 2013), table tennis (Rodrigues et al. 2002) and baseball (Bahill and LaRitz 1984) show that the eye movements that people make are tuned to the ball’s flight. One may wonder whether the way people direct their eyes is only aimed at increasing the visual resolution (Bock 1993; Prablanc et al. 1979) and improving velocity judgments (Brenner and Smeets 2011, 2015; de la Malla et al. 2017), or whether it is also easier to guide the hand towards where one is looking (Ballard et al. 1992; Bekkering and Sailer 2002; Gowen and Miall 2006).

People do not necessarily move their arm towards where they are looking, because they pursue a target with their eyes when manually tracking it with delayed visual feedback about the movement of their hand, despite the hand being systematically ahead of the target (Vercher and Gauthier 1992). Introducing delays between tapping movements of the hand and vision of the tapping hand is known to lead to adaptation of the judged relative timing between the action and the visual feedback (Keetels and Vroomen 2012). Such adaptation may also play a role when tracking or intercepting moving targets with delayed feedback, because it may make one judge the hand to be at a position that it has actually already passed some time ago. Alternatively, in an interception task, people might look ahead of the target and move towards where they look to compensate for the delay. Moreover, considering that gaze is generally directed towards positions at which acquiring information is most beneficial (Brenner and Smeets 2011), we might expect gaze to be directed towards the visual feedback of the hand if there is a delay between the unseen hand and such feedback.

To evaluate these possibilities, we designed two experiments in which participants had to intercept moving targets with their finger. In some conditions, they did not see their finger but saw a cursor that followed their finger movement with a delay. In the first experiment, we examined whether gaze would be directed ahead of the target to compensate for a predictable delay between the finger and the cursor. In the second experiment, we examined whether gaze would be directed at the cursor when the delay was not predictable. In both cases, participants kept their eyes on the targets and were quite successful in intercepting the targets.

## General methods

**Fig. 1:**
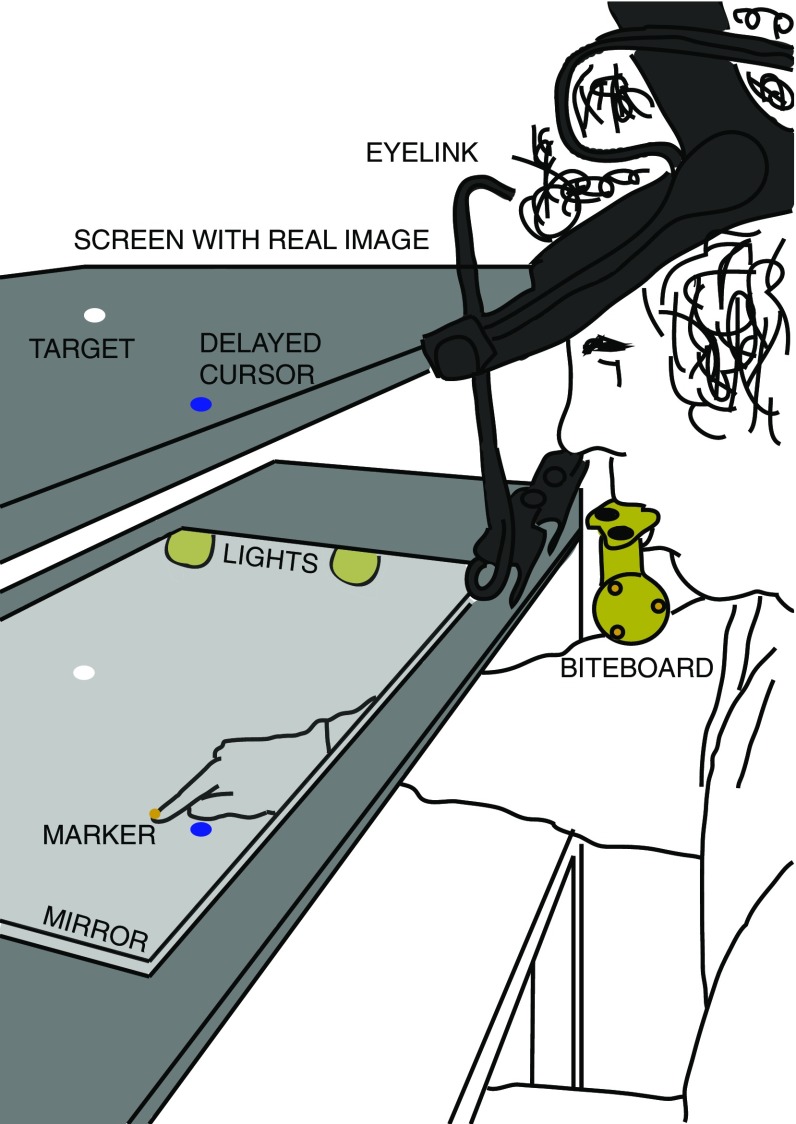
Schematic representation of the setup. Images were projected onto a screen located above a half-silver mirror, so that the target (and sometimes a cursor) appeared to move across a surface below the mirror. Lights below the mirror controlled whether or not the hand was visible: it was visible when the lights were on but not when they were off

Participants sat in front of a horizontal surface (Fig. 1) and looked into a half-silvered mirror above that surface. Stimuli were projected from above onto a horizontal back-projection screen above the half-silvered mirror, creating the illusion that the stimuli were in the same plane as the surface below the mirror. Participants’ task was to slide their finger across the surface below the mirror to intercept a laterally moving target. Lights below the half-silvered mirror controlled the visibility of the hand.

Two Optotrak 3020 systems were used to record the position of the index finger and the position and orientation of the head (at 250 Hz). One of the Optotrak systems was placed behind the setup from the participant’s perspective to track the position of a marker attached to the nail of the index finger of the participant’s dominant hand. The second Optotrak system was placed to the left of the setup to track the positions of three markers attached to a bite-board that the participants held in their mouth, but that was not fixed in space so that participants could move their heads freely. The participant’s eye movements were recorded with an Eyelink II system at 500 Hz. The positions of the finger with respect to the image on the screen and of the eyes with respect to the markers on the bite-board were calibrated at the beginning of each session (for details of the calibration see (de la Malla et al. 2017). Gaze and finger trajectories were determined from the measured Optotrak and Eyelink data during all trials (accounting for head movements on the basis of measurements of the position of the bite-board). The minimal delay between a movement and adjustments to the image in response to such movements was about 59 ms (it varies a bit because the rate and timing of the Optotrak measurements were not synchronized with the frame rate of the projector; 60 Hz; 800 ${\times }$ 600 pixels; 61 ${\times }$ 46 cm^2^).

### Procedure

Participants were instructed to intercept a laterally moving target by passing through it. They were asked to do so in a single continuous movement. To start a trial, participants had to place their index finger on a green, 1.4-cm-diameter, static disk that was at the centre of the screen laterally, and 20 cm closer to the participant than the target’s path. The target was a white, 2-cm-diameter disk that moved laterally at a constant velocity. Participants were free to start moving whenever they liked and to hit the target when and wherever they wanted. They could take breaks whenever they wanted by simply not moving to the starting position. When lights below the mirror were on, participants could see their hand. When they were off, they could not. In the latter case, they could sometimes see a cursor that presented delayed visual feedback about the position of the finger. The cursor was a blue, 8-mm-diameter disk. Details of the conditions are presented separately for each experiment.

### Analysis

Trials in which participants lifted their finger from the surface during the interceptive movement or did not reach the target’s path were excluded from the analysis (fewer than 2% of the trials). The two eyes’ gaze trajectories on the screen (as determined by combining the Eyelink data with information about the position and orientation of the head) were averaged and then filtered with a low-pass Butterworth filter with a cut-off frequency of 100 Hz (applied in both directions to maintain synchrony with the other measurements). Velocities of movements of the finger and gaze were computed by dividing the distance between the (interpolated) positions 15 ms before and after each moment by the corresponding time interval of 30 ms. The finger was considered to have started moving when its velocity in the sagittal direction reached 3 cm/s.

**Fig. 2:**
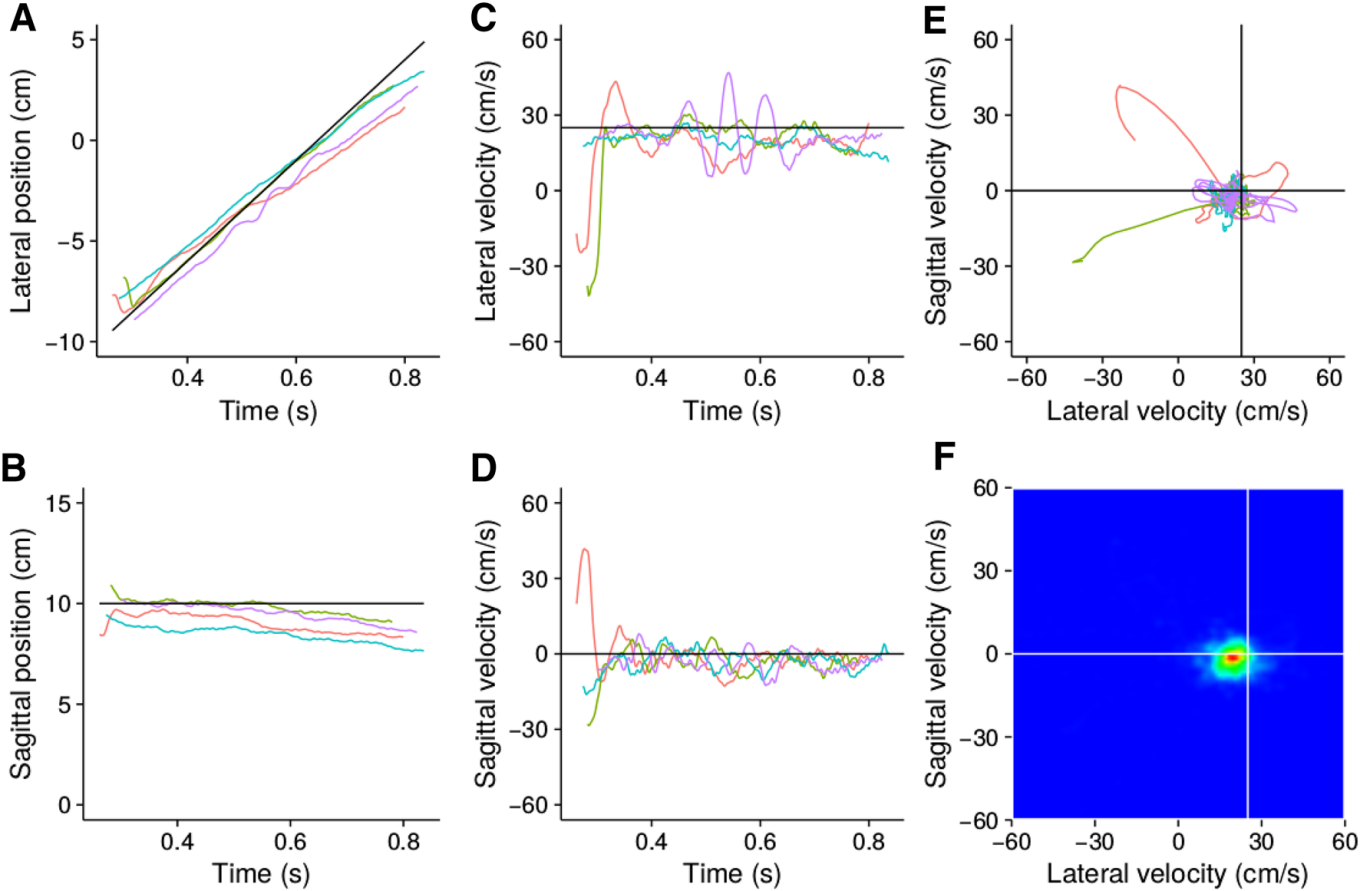
Step by step explanation of the gaze analysis. Lateral (**a**) and sagittal (**b**) gaze positions across time are shown for 4 representative trials of one condition (colour coded). The corresponding velocities are shown both as a function of time (**c**, **d**) and relative to each other (**e**). The latter can be converted into a heat map (**f**). This heat map shows information for more such trials than these four. Density was normalized so that the most frequent combination of velocities is represented by pure red and ones that did not occur are represented in pure blue. Black lines in the plots and white ones in the heat map represent the target values. The pursuit gain in these examples is lower than 1

To provide a concise representation of gaze behaviour, we made heat maps of the combination of sagittal and lateral gaze velocities from the moment the finger (or the cursor whenever a cursor was shown) started moving until when the finger crossed the target’s path. The steps that were taken to obtain the heat maps are shown in Fig. 2. Figure 2a and b shows how the lateral and sagittal gaze positions change across time for four trials (colour coded) from the moment when the cursor started moving until when the finger crossed the target’s path. Figure 2c and d shows the gaze velocities for the same trials. Plotting the combination of lateral and sagittal velocities at each moment, rather than each as a function of time, summarizes how gaze is changing during these trials, confirming that the eyes are generally pursuing the target (Fig. 2e). The heat map in Fig. 2f shows similar data to that in Fig. 2e, but while drawing all individual traces becomes very messy when more trials are included, this way of presenting the data becomes clearer with more data. The colour indicates the relative frequency of occurrence of each combination of velocities.

The temporal error (de la Malla et al. 2014) was used to evaluate the degree of adaptation to the delay. This value is the difference between the lateral positions of the target and of the finger at the time at which the finger crosses the target’s path, divided by the velocity of the target. If participants do not adapt to the delay, so that they intercept the target with their (unseen) finger, the temporal error is zero. If participants hit the target with the delayed cursor, the temporal error is equal to the imposed delay between the finger and the cursor.

To determine whether the eyes were directed at the target or ahead of the target, the temporal ’error’ of gaze (Eq. 1) was computed in the same way as the temporal error of the hand:

1 $$\begin{aligned} t_\mathrm{err}^\mathrm{Gaze}(X) = \frac{X_\mathrm{e} - X_\mathrm{T}}{v_\mathrm{T}}, \end{aligned}$$

where $X_\mathrm{e}$ refers to the lateral eye position, and $X_\mathrm{T}$ and $v_\mathrm{T}$ are the lateral position and velocity of the target, respectively. Possible drifts in the eye movement recordings, for instance as a result of headband slip, can give rise to small systematic lateral errors. In experiment 1, in which targets moved to the left and to the right on different trials, such errors cannot systematically influence the mean temporal error, because if they increase the temporal error for targets moving in one direction they will decrease it for targets moving in the opposite direction. However, because such errors do increase the variability, we corrected to some extent for such drifts on the basis of where participants were looking when the target appeared. The median of the lateral gaze position at the moment that the target appeared was determined for the 5 trials closest in time to the trial in question, and the deviation of this value from the screen centre was subtracted from the lateral gaze position before determining the temporal error. This method introduces some variability, because participants were not obliged to look at the screen centre when the target appeared, but since the starting point was centred and the target could appear at either side, there was no reason to look in a particular lateral direction. Comparing individual subjects’ data with and without this correction showed that applying this correction generally reduced the variability a bit, so we applied the correction to all the data. In experiment 2, we could not correct the data in this way because all the targets were moving from left to right. The temporal error of the gaze was measured 100 ms before the finger crossed the target’s path, which is the last moment at which sensory information can be used to guide the finger (Brenner and Smeets 2003; de la Malla et al. 2012; López-Moliner et al. 2010).

## Experiment 1

The main question of this first experiment was whether gaze behaviour would change when feedback about the on-going movement of the finger is delayed. We recorded both gaze and finger movements while participants intercepted moving targets. We did so when the hand was visible, when there was no visual feedback about the hand movement, and when a cursor presented delayed feedback about the position of the finger. We evaluated the extent to which participants had adapted to the delay by comparing the movements during trials without visual feedback before and after the exposure to the delayed feedback. Details of the trials are provided below.

The hypothesis that we test in this experiment is that gaze is directed ahead of the target that one is trying to intercept if there is a predictable delay between the cursor and the finger. If participants do so, the temporal error in their gaze (how far ahead of the target they are looking; see “Analysis”) should match the delay between the cursor and the finger. In that case, participants could hit the target by moving their finger to intercept their gaze trajectory.

### Participants

A total of 13 participants (8 females; ages between 26 and 59) took part in this experiment at the Vrije Universiteit Amsterdam, including 3 of the authors. All participants had normal or corrected-to-normal vision (3 participants wore contact lenses and 3 wore glasses). One participant was left handed. None had evident motor abnormalities. All participants were members of the department of Human Movement Sciences and gave their written, informed consent before taking part in the experiment. Except for the authors, participants were unaware of the purpose of the experiment.

### Conditions

The target appeared 15 or 17 cm to the left or to the right of the lateral midline of the screen and moved at 20 or 30 cm/s laterally towards and then past the midline. There was a single session of 271 trials that took around 25 min to complete. During the first 40 trials, the light beneath the mirror was on so that participants had full vision of their hand (hand condition). During the next 40 trials, the light was off and no other visual feedback was provided (no feedback condition). During the next 151 trials the light was off but visual feedback about the position of the finger was provided in the form of a cursor (a blue, 8-mm-diameter disk). On the first ten trials, the position of the cursor followed that of the finger with a delay of about 59 ms (the minimal delay we could achieve with our setup). After that, the delay increased by 1 ms per trial until it reached 200 ms (adaptation condition). We increased the delay gradually to stimulate a smooth adaptation (de la Malla et al. 2014; Honda et al. 2012). Finally, during the last 40 trials there was no visual feedback again (no feedback 2 condition). For the hand, no feedback and no feedback 2 conditions, there were 5 trials with each of the 8 combinations of starting position and velocity. They were presented in random order within each condition. In the adaptation condition, the combination of starting position and velocity was chosen at random on each trial.

Each trial started by participants placing their finger at the starting position. In the hand condition, they could see their hand when moving to the starting position. In the other conditions, we did not want to expose participants to a delay (or absence of delay) before the movement started. However, we had to help them find the starting position. We did so by displaying a static cursor at the position of the finger whenever the finger stopped moving (when it moved less than 0.04 mm in 4 ms). This was sufficient information to comfortably guide the unseen finger to the starting position. Once the finger was at the starting position for a random period between 600 and 1200 ms the trial started: the moving target appeared.

### Results

**Fig. 3:**
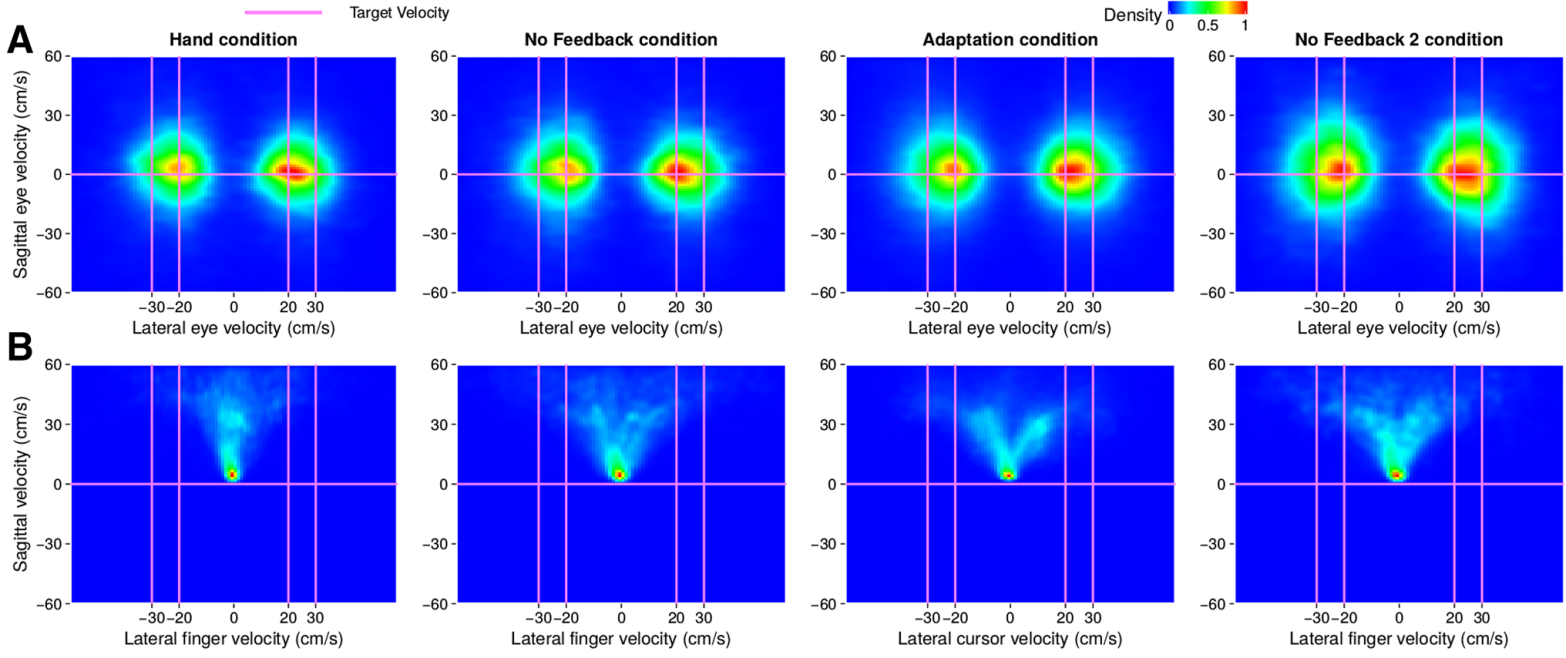
Heat maps of the occurrence of lateral and sagittal velocities of eye movements (**a**) and of finger movements (or cursor movements in the adaptation condition) (**b**) in Experiment 1. Each column corresponds to a different condition. The intersections of the pink lines indicate the four target velocities. Only the time between when the finger (or cursor in the adaptation condition) starts moving (velocity threshold of 3 cm/s) and when the finger (in all conditions) crosses the targets path is considered. Density was normalized for each panel: all values within each panel were divided by the maximal value within that panel

Gaze was primarily directed at the target rather than at the finger or the cursor. This can be seen by looking at the velocity of the gaze (Fig. 3a) that corresponds reasonably well with the targets’ velocities (intersections of pink lines) independently of how the finger (and cursor in the adaptation condition) moved (Fig. 3b). Gaze is expressed as a velocity on the screen, rather than as an angular velocity, because doing so makes it easier to relate gaze to motion of the cursor and target, especially since participants were free to stand wherever they liked. Since the freedom to move was restricted by having to be able to reach the targets, 1 cm/s of gaze velocity corresponds to a rotational velocity of about 1${^\circ }$/s. Participants generally made saccades to the targets before they started moving their finger. There were very few saccades that displaced gaze by more than 10 cm while the finger was moving.

**Fig. 4:**
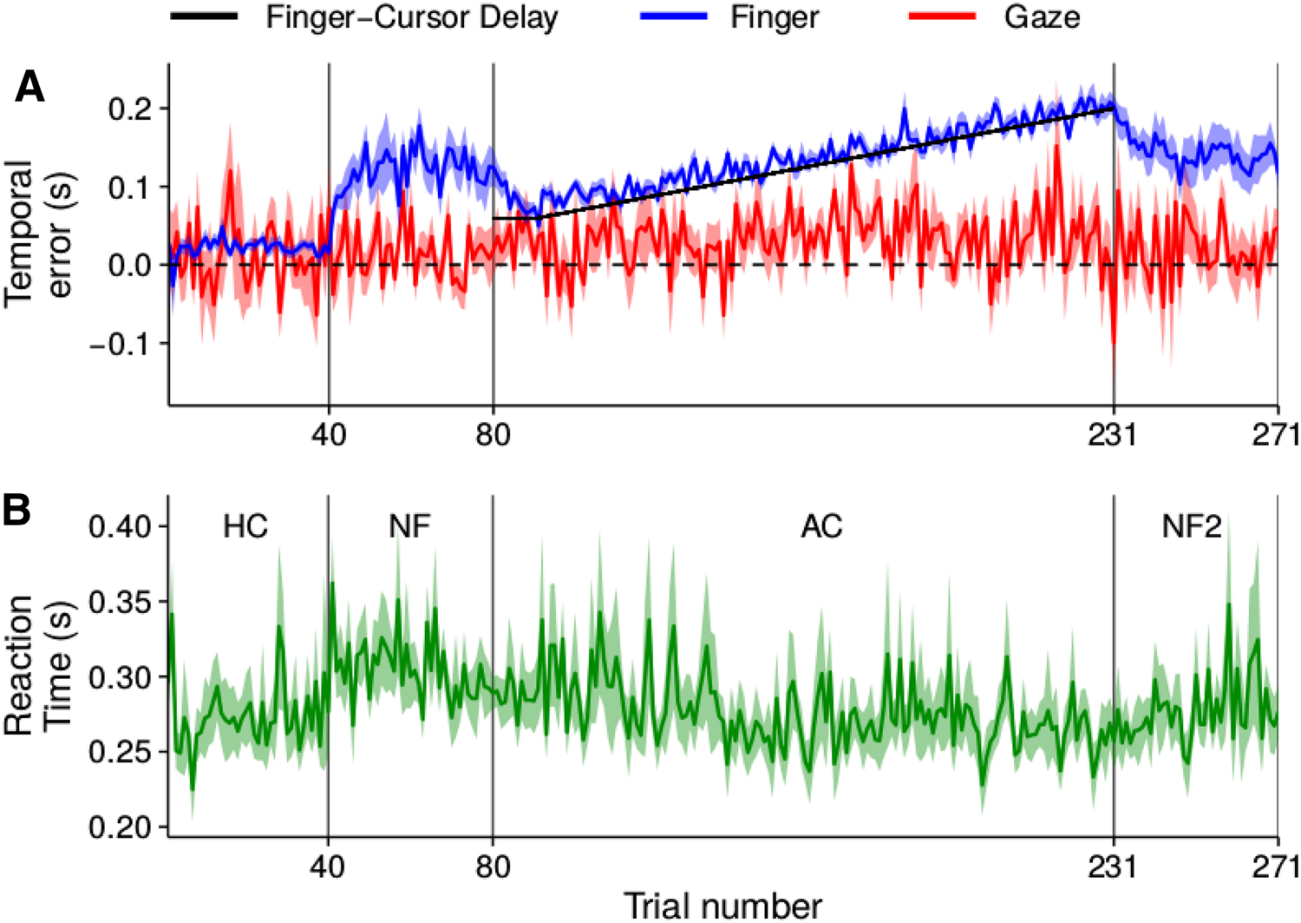
Temporal errors of the finger and gaze (**a**) and reaction times (**b**) on consecutive trials. Lines depict the average of all participants for each trial, with the shaded areas showing the standard error across participants. The black line indicates the gradually increasingly delay between the finger and the cursor. The temporal error of the finger (blue) is determined at the moment that the finger crosses the targets path. The temporal error of gaze (red) is determined 100ms before that. The reaction time (green) is determined with a threshold velocity of the finger of 3 cm/s. The vertical grey lines segregate the 4 conditions: *HC* hand condition, *NF* no feedback condition, *AC* adaptation condition, *NF2* no feedback 2 condition

There appeared to be a slight tendency to intercept the target ahead of its centre when the hand was visible (blue curve reveals a slightly positive temporal error during the first 40 trials in Fig. 4a). This tendency increased to more than 100 ms when feedback was removed (trials 41–80). When feedback was provided in the form of a delayed cursor (trials 81–231), the error between the finger and the target matches the value of the imposed delay (black line) quite precisely, meaning that participants tried to hit the target with the cursor rather than with the finger. When the cursor was removed, the error gradually returned to its previous value when there was no feedback (an error of slightly more than 100 ms; trials 231–271). Unlike the hand, the temporal error of the gaze (red curve) did not seem to be affected by the feedback about the on-going movement of the finger. The temporal error of the gaze was clearly closer to zero (dashed line) than to the imposed delay (black line). Thus, participants were looking close to the target centre (temporal error of zero) rather than ahead of the target towards where their finger would have to pass for the delayed cursor to hit the target (temporal error equal to the delay, which is up to 6 cm ahead of targets moving at 30 cm/s and 4 cm ahead of targets moving at 20 cm/s, because the maximal delay is 200 ms).

One way to deal with a delay between one’s actions and their consequences would be to start moving sooner. Figure 4b shows that participants did start to move their finger earlier as the delay increased in the adaptation condition. However, the change is only a fraction of the difference in delay. The delay in the adaptation condition increased at 1 ms/trial whereas the reaction time only decreased at about 0.23 ms/trial. Moreover, the latter decrease might represent returning to the normal value with feedback (as in the hand condition). Table 1 shows the average peak velocity of the fingers’ interceptive movement across the surface, the time to this peak velocity, and the total time until the finger crossed the target’s path, for each of the conditions. Given that participants gradually adapted to the imposed delay (blue curve in Fig. 4a) in the adaptation condition, only the values for the first and last 10 trials of this condition are shown (separately). The finger moved faster when there was no visual feedback, compensating to some extent for the longer reaction time [as in Carson et al. (1993), Elliott et al. (1991)].

**Table 1:** Peak velocity of the finger, time to peak velocity of the finger, and time it took for the finger to reach the target’s path for each condition (mean ± SE across participants)

Condition	Peak velocity (cm/s)	Time to peak velocity (ms)	Total time (ms)
HC	69 ± 5	645 ± 44	771 ± 44
NF	79 ± 7	691 ± 41	782 ± 41
AC (first 10)	67 ± 5	691 ± 43	845 ± 43
AC (last 10)	69 ± 7	633 ± 52	817 ± 59
NF2	76 ± 7	675 ± 43	774 ± 47

## Experiment 2

Experiment 1 reveals that participants did not look ahead of the target to compensate for the delay between the cursor and the finger, but always kept their eyes on the target. Participants did not simply rely on haptic information about the position of their index finger, because they adjusted its movements to intercept the target with the cursor in the adaptation condition. They did not only do so by guiding the visible cursor to the target during the trials, because the adaptation clearly influenced the first trials after the feedback was removed. Perhaps the adaptation proceeded so automatically that it was more useful to visually monitor the target’s movement than to monitor the gradual progress of the delay between the finger and the cursor by looking at the cursor.

To examine whether participants would look at the cursor if the cursor’s behaviour were less predictable, in which case seeing how it moves provides more useful information, we conducted a second experiment in which we randomly varied the delay between the finger and the cursor across trials. We also made the behaviour of the target completely predictable by not varying the direction and velocity of its motion. Note that randomly varying the delay means that participants can adapt to the average delay, but cannot adapt in a manner that gives rise to adequate differences in performance between trials with different delays. Such differences can only result from adjusting on-going movements.

The hypothesis that we test in this experiment is that randomly varying the delay from trial to trial will make participants look at the delayed cursor to judge by how much the cursor is delayed with respect to the finger on that particular trial.

### Participants

A total of nine participants (seven females; ages between 28 and 59) took part in the second experiment at the Vrije Universiteit Amsterdam. Seven of them had taken part in the former experiment, including two of the authors. All participants had normal or corrected-to-normal vision (one participant wore contact lenses and three wore glasses). One participant was left handed. None had evident motor abnormalities. All participants were members of the department of Human Movement Sciences and gave written, informed consent before taking part in the experiments. Except for the authors, participants were unaware of the purpose of the experiment.

### Conditions

There was a single session of 260 trials that took around 20 min to complete. The lights beneath the half-silvered mirror were off and a cursor provided visual feedback about the on-going movement of the finger as in the adaptation condition of Experiment 1. The target always moved from left to right at 25 cm/s, starting 16 cm from the lateral midline. The first 20 trials had the minimal delay of about 59 ms (D59 condition). The next 80 trials had a delay of 200 ms (D200 condition). The remaining 160 trials had delay values of 59, 100, 150 and 200 ms (random delay condition). There were 40 trials for each delay, and the trials were presented in random order.

### Results

**Fig. 5:**
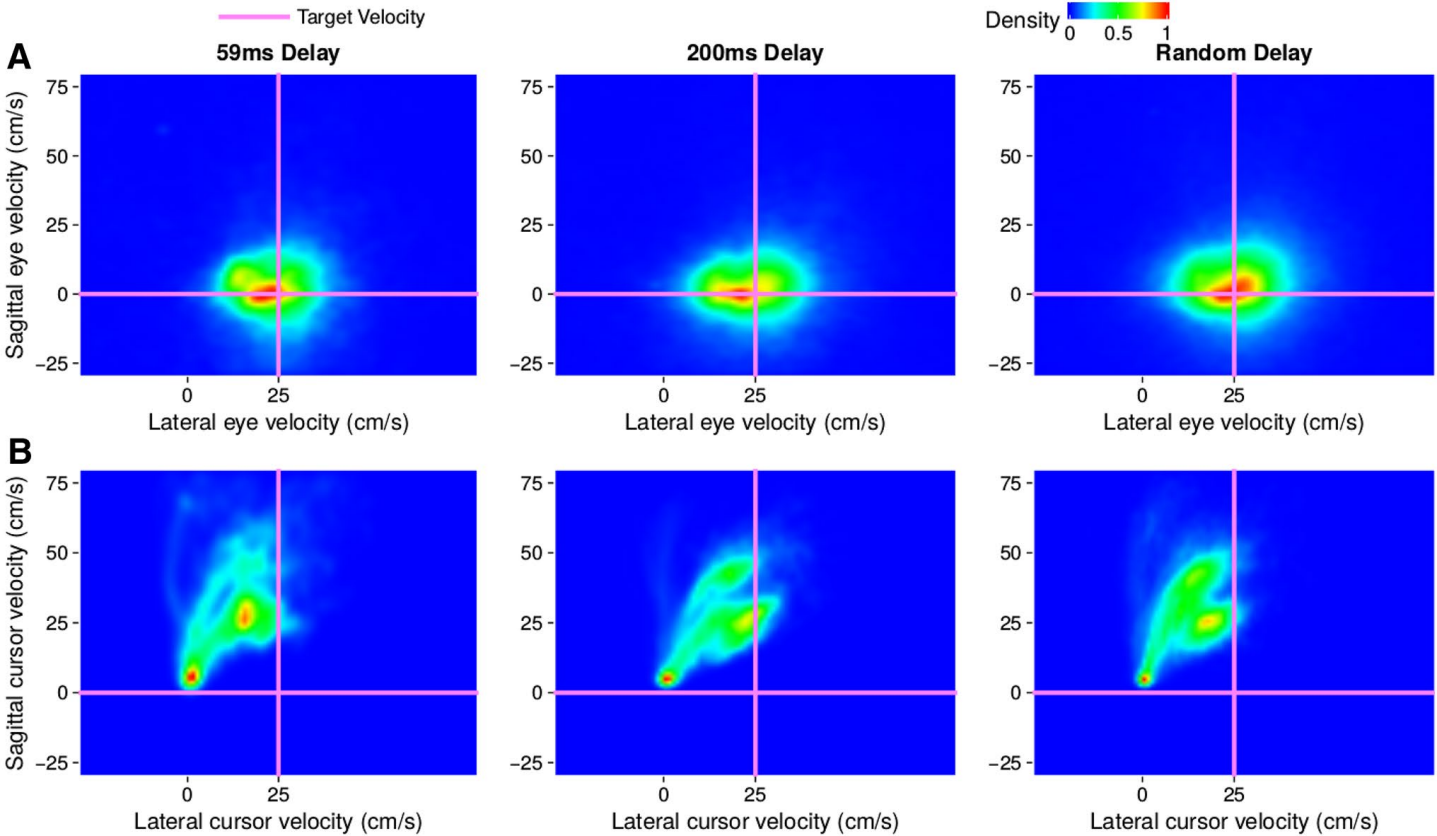
Heat maps of the occurrence of lateral and sagittal velocities of eye movements (**a**) and of cursor movements (**b**) in Experiment 2, for all participants except for participant 6. Each column corresponds to a different condition. Pink lines indicate the target velocity. Only the time between when the cursor starts moving (velocity threshold of 3 cm/s) and when the finger crosses the target’s path is considered. Density was normalized for each panel: all values within each panel were divided by the maximal value within that panel

**Fig. 6:**
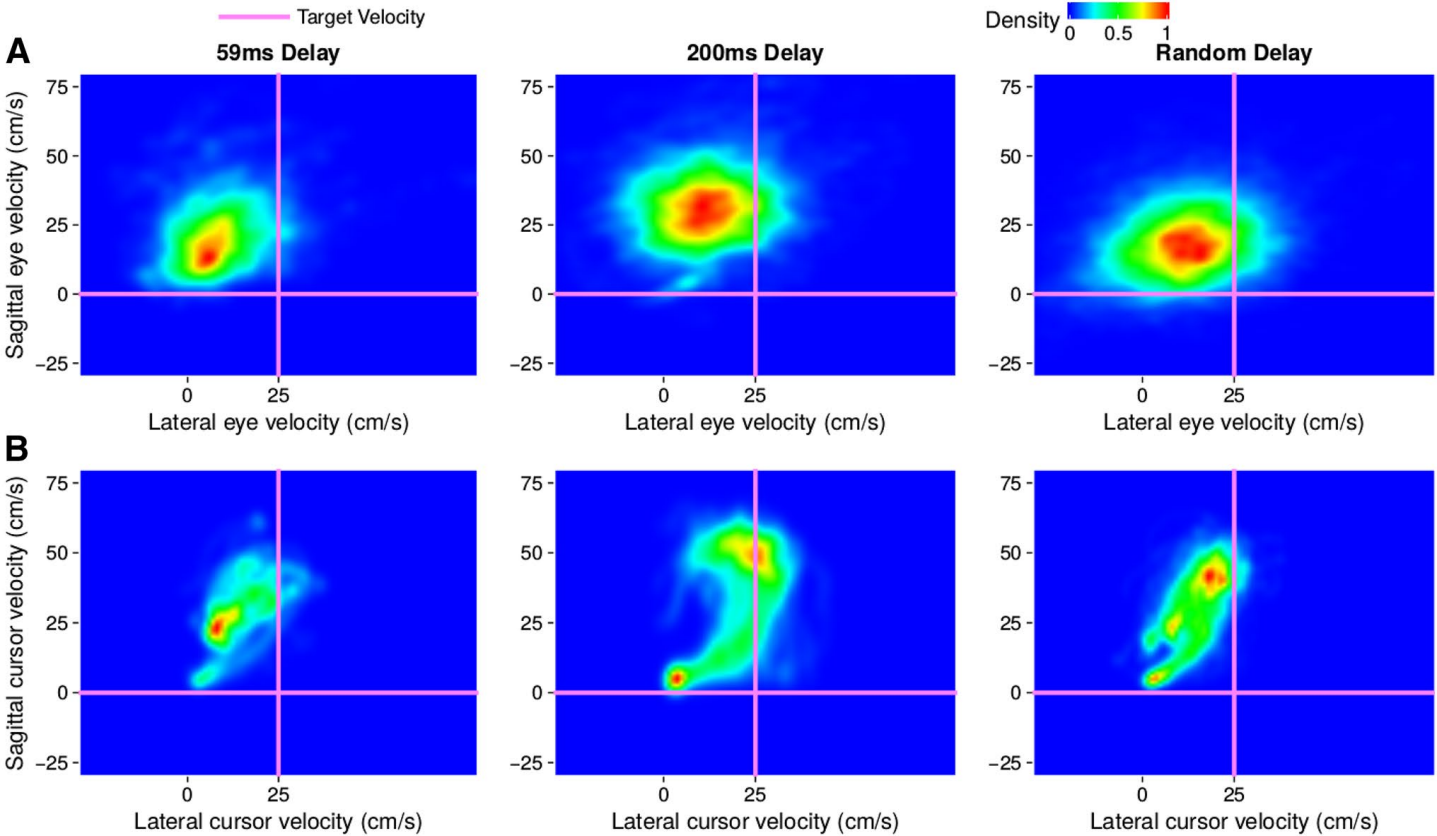
Heat maps of the occurrence of lateral and sagittal velocities of eye movements (**a**) and of cursor movements (**b**) for participant 6 in Experiment 2. Details as in Fig. 5

**Fig. 7:**
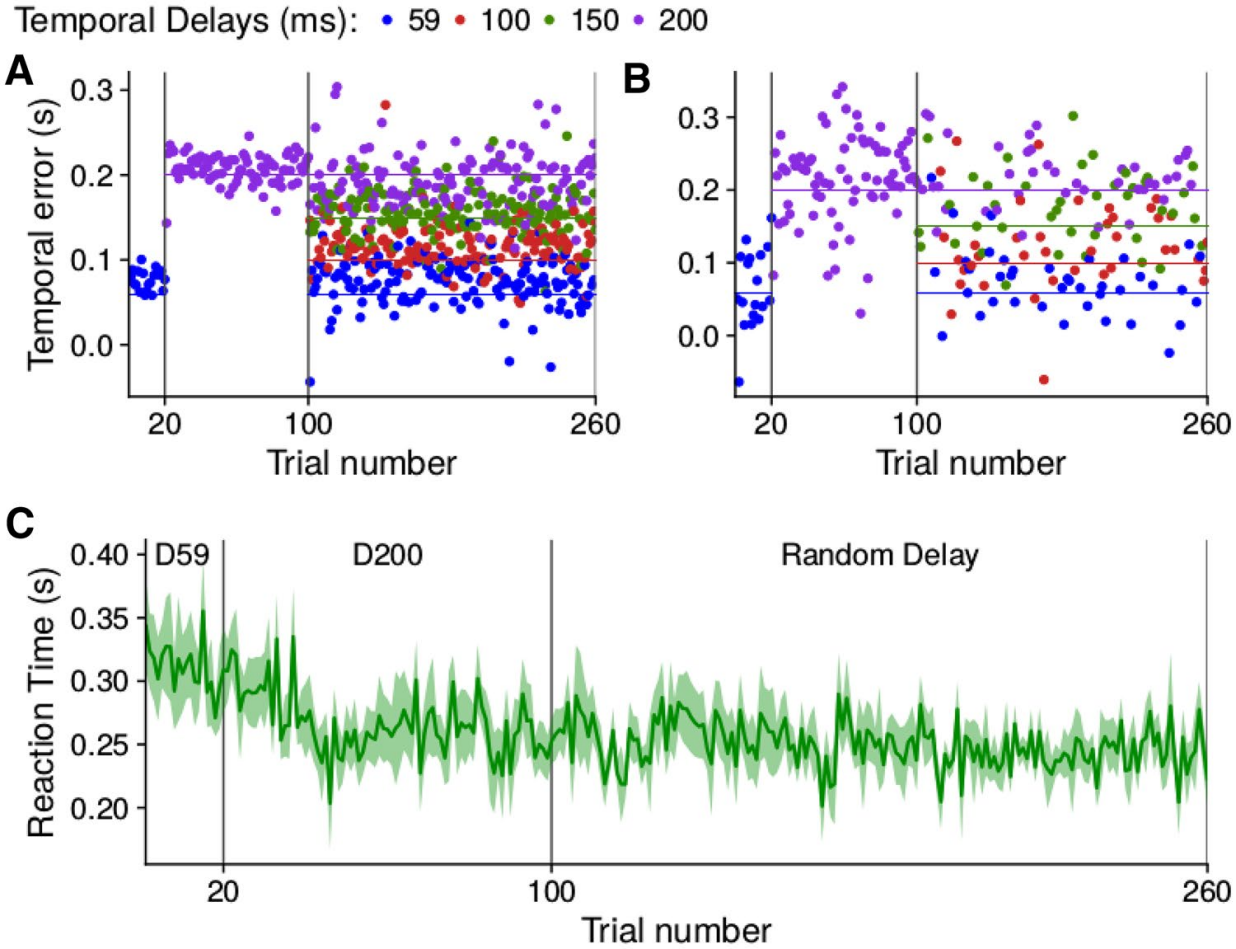
Temporal errors of the finger (**a**, **b**) and reaction times (**c**) on consecutive trials. Temporal errors are shown separately for participant 6 (**b**) and the average of all other participants (**a**). In the random delay condition of the latter case, each point shows the average value of the temporal error of all participants that were subjected to that temporal delay on that trial. The horizontal lines in **a** and **b** indicate the temporal error that will make the cursor hit the target when the cursor is delayed by 59, 100, 150 or 200 ms (as indicated by their colour). Reaction times are averaged across all participants. Vertical lines segregate the three conditions: 59 ms Delay (D59), 200 ms Delay (D200) and random delay. Other details as in Fig. 4

Since one participant (participant 6) had a different gaze pattern than all the others, this participant is treated separately. The other participants showed the same gaze pattern as in Experiment 1. Even when the delay varied randomly across trials (rightmost panels in Fig. 5) they directed their gaze towards the target (Fig. 5a) rather than the cursor (Fig. 5b). Participant 6 might have pursued the cursor to some extent (Fig. 6). Despite this difference in gaze behaviour, the temporal errors of the other participants (Fig. 7a) were quite similar to those of participant 6 (Fig. 7b). The finger moved in a manner that ensured that the cursor intercepted the target (points close to the lines of the same colour in Fig. 7a). We used a repeated measures analysis of variance to evaluate whether the temporal error was influenced by the delay in the random delay condition. This analysis was based on the mean temporal error per participant for each delay. The analysis shows that the temporal error depends on the delay: *F*[3,21]=57 $p<$ 0.0001. Note that the fact that the data appear to be more variable in Fig. 7b than in Fig. 7a is mainly because the value for each trial corresponds to the average of the temporal error of eight participants for conditions D59 and D200 in Fig. 7a, whereas it is the single value of participant 6 in Fig. 7b. In the random delay condition of Fig. 7a the number of trials that are averaged varies, because the four temporal delays were presented in random order, and values for each trial number were averaged across participants for each delay. It is important to remember that the temporal error is defined with respect to the finger, so that an error equivalent to the delay is required to hit the target with the cursor.

**Table 2:** The peak velocity and time to peak velocity of the finger, and the time it took the finger to reach the target’s path (mean ± SE across participants)

Condition	Peak velocity(cm/s)	Time to peak velocity (ms)	Total time (ms)
Blocked 59	63 ± 6	724 ± 69	906 ± 61
Blocked 200	62 ± 5	644 ± 70	860 ± 70
Random 59	54 ± 4	591 ± 57	882 ± 57
Random 100	56 ± 4	605 ± 57	860 ± 57
Random 150	57 ± 4	639 ± 61	848 ± 53
Random 200	59 ± 5	660 ± 64	842 ± 55

The reaction time (Fig. 7c) appears to gradually decrease during the first 50 trials and to then remain stable at about 254 ms. The reaction time cannot depend on the delay in the random delay condition, because the delay can only be known once the movement has started, so we averaged across the delays. The peak velocity of the finger appears to be slightly lower when the delays were presented in random order than when they were blocked (Table 2), perhaps because participants realized that they had to rely more on guidance during the movement when the delay was unknown until they started moving. There also appeared to be a tendency to move faster for longer delays in the random delay condition, presumably to compensate for the delay. In accordance with the higher peak velocity for longer delays in the random delay condition, the time to peak velocity did not increase by as much as the delay. The total time taken for the finger to reach the target’s path also appeared to be shorter when the delay was longer.

## Discussion

In accordance with the previous studies (de la Malla et al. 2014; Honda et al. 2012), participants adapted their movements to the delays that were imposed between the moving finger and a cursor representing that finger. We here show that such adaptation does not involve a change in gaze patterns. In general, the eyes pursue the target smoothly with a gain that is somewhat variable and slightly lower than one (as also shown in de la Malla et al. 2017). Participants did not look ahead of the target, so that they could move their finger towards where they were looking to compensate for the delay (Fig. 4a). Neither did the delay make them pursue the cursor with their eyes rather than pursuing the target (Figs. 3 and 5), except perhaps for one of the nine participants of Experiment 2 (Fig. 6). Somewhat surprisingly, this participant appears to have followed the trajectory of the cursor from the beginning of the session, rather than only when the delay varied across trials (Fig. 6). This participant’s gaze pattern does not closely match that of the cursor, so it is also possible that something went wrong with the eye movement recordings, but we have no independent evidence of this. Since all participants’ temporal errors were tuned to the delay on each trial, even when they could not know the delay until they started moving (Fig. 7a, b), the participants who were pursuing the target with their eyes must have been using peripheral vision to guide the cursor to the target, rather than only relying on adaptation to the delay on the basis of feedback from previous trials.

It is somewhat surprising, considering that people usually look where the most critical information is to be found, that most participants kept their eyes on the target that was moving in a completely predictable manner, and relied on peripheral vision to guide the relatively small cursor towards the target. Note that this cannot be because of any advantage of moving the finger towards where they are looking (Prablanc et al. 1979), because they are guiding the cursor to the target that they are pursuing by moving their finger well ahead of the target. It also cannot be because they need to know the target’s position more precisely (Brenner and Smeets 2011, 2015), because the important aspect in terms of hitting the target is the relative position between the two. Possibly, peripheral vision of the cursor is combined with haptic information about the position of the finger, which might explain why the temporal errors do not quite match the delays when they are not predictable (Fig. 7a). When they are predictable, or when they change very gradually (Fig. 4a), adaptation to the delays probably improves performance. Similarly, looking at the cursor (Fig. 7b) might improve performance, presumably because vision is given more weight than haptics when one is looking at the cursor. However, although the errors look more systematic in the right side of Fig. 7a than in Fig. 7b or Fig. 4a, we would need to find more than one subject who pursues the cursor to draw any real conclusions from such differences. Note that the systematic biases are towards the mean delay, rather than towards no delay, suggesting that adaptation has taken place, despite using peripheral vision to guide the cursor to the target.

Our results show that people keep their gaze on the target that they are trying to intercept, even if they have to intercept the target with delayed visual feedback of their on-going movement. Most people did not even divert their gaze from the target when we tried to make it advantageous to look elsewhere by varying the delay and not the target motion (second experiment). Thus, we can conclude that having to deal with delays does not influence people’s tendency to look at objects with which they intend to interact. People did adapt to the delay, and they used peripheral vision to guide the delayed visual representation of their hand to the target, rather than bringing their hand to the target or to where they were looking. Presumably, adaptation to constant delays and guiding relevant items movements in the manner that we observed is what allows us to use the many electronic devices that we so enjoy.
